# Epidemic cholera in the new world: translating field epidemiology into new prevention strategies.

**DOI:** 10.3201/eid0104.950408

**Published:** 1995

**Authors:** R V Tauxe, E D Mintz, R E Quick

**Affiliations:** National Center for Infectious Diseases, Centers for Disease Control and Prevention, Atlanta, Georgia, USA.


### Epidemic Cholera in the New World: Translating Field Epidemiology into New Prevention Strategies

Cholera, a devastating diarrheal disease, has swept through the world in recurrent pandemics since 1817. The seventh and ongoing pandemic began in 1961 when the El Tor biotype of *Vibrio cholerae* O1 emerged in Indonesia. This pandemic spread through Asia and Africa and finally reached Latin America early in 1991 (1). After explosive epidemics in coastal Peru, it spread rapidly and continues throughout Latin America (Figure 1). Because of underreporting, the more than 1,000,000 cholera cases and 10,000 deaths reported from Latin America through 1994 (Table 1) (2) represent only a small fraction of the actual number of infections. Molecular characterization of *V. cholerae* O1 strains from Peru has shown that they do not match strains from anywhere else in the world; therefore, the source of the Peruvian epidemic strains remains unknown (3). Moreover, other strains have since appeared in Latin America. At least one of these, a strain resistant to multiple antimicrobial drugs, was first identified in Mexico and elsewhere in the world in mid-1991 and has since spread widely throughout Central America (4). The introduction of strains into new areas illustrates the rapid global transfer of pathogens. *V. cholerae* O139 Bengal, which emerged as a new cause of epidemic cholera in Asia in 1992, could also appear in Latin America (5).

Such introductions are not easy to prevent, because they may follow the arrival of travelers who are not aware of their infection or of ships carrying contaminated ballast water. The key to controlling epidemic cholera lies in limiting its spread by using measures that prevent sustained transmission. One measure might be using an inexpensive and effective vaccine to provide lasting protection; however, no such vaccine yet exists, although progress in vaccine development is being made (6-8). Another measure is interrupting transmission so that the causative organism never reaches the human host. This approach to prevention successfully controlled many epidemic diseases in the industrialized world, including cholera, typhoid fever, plague, and malaria, before vaccines or antibiotics were developed. Over the last century, a large engineering infrastructure, built in industrialized nations, has provided safe water and sewage treatment for nearly all people in these nations and has made sustained transmission of cholera in those countries extremely unlikely. Despite sporadic cases along the U.S. Gulf Coast and repeated introduction of the epidemic organisms by travelers, epidemic cholera has not occurred in the United States since the nineteenth century (9,10).

To prevent cholera by interrupting transmission of the organism to the host, it is important to understand precisely how the bacteria are transmitted. John Snow demonstrated waterborne transmission of cholera during a large epidemic in London in 1856 (11). He and many others since have suspected that other routes of transmission are also important. Epidemiologic investigations during the seventh pandemic have documented a variety of specific food and water pathways by which the bacteria reach the host, some of which were new and unsuspected (12). The El Tor biotype of *V. cholerae* O1, for example, multiplies rapidly in moist foods of neutral acidity (13). This bacterium also persists in the estuarine environment in niches that are poorly understood but may involve the plankton on which shellfish feed. This means

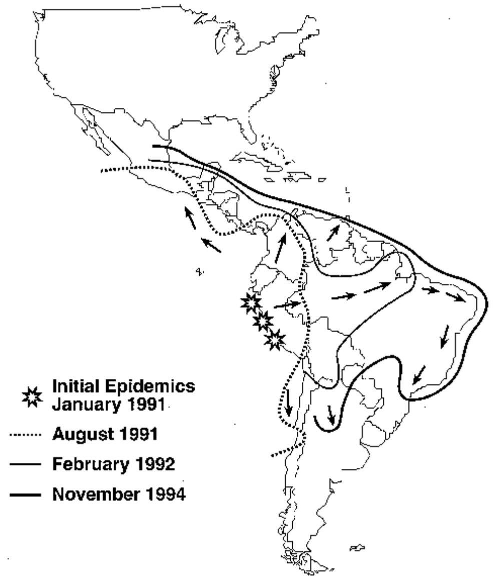

Figure 1. Geographic extent of the Latin American cholera epidemic over time. Lines represent the advancing front of the epidemic at different dates. As of mid-1995, all Latin American countries except Uruguay have reported cases; no cases have been reported from the Caribbean.

that raw seafood can be contaminated naturally before it is harvested. Understanding these pathways of transmission in detail has been central to devising successful control measures to block them. For example, advice to drink only boiled or bottled water would be of little use in outbreaks where the source was actually contaminated food, such as shellfish or leftover rice. On at least one occasion, such advice actually worsened the situation because the bottled water was itself contaminated (14).

When epidemic cholera appeared in Latin America, after an absence of more than 100 years, we conducted a series of eight rapid field investigations in collaboration with national public health authorities and the Pan-American Health Organization to define the pathways of disease transmission and the priorities for prevention. Conducted in various settings between February 1991 and August 1993, these investigations guided the initial emergency prevention efforts and the development of sustained prevention measures (Table 2) (15-21).

The same case-control method was used for each investigation. We first interviewed a few patients in great detail about what they had ingested in the 3 days before they became ill, probing for known or potential vehicles of cholera. We then constructed a standardized interview questionnaire that asked about possible exposures. Using this questionnaire, we interviewed patients recovering from cholera as well as healthy persons of the same age and sex who lived in the same neighborhood. By comparing the frequency of positive and negative responses among the ill and well persons, we could identify the exposures most strongly associated with disease. For example, if 25 of 35 patients, but only 5 of 35 matched controls, reported eating sliced mango from street vendors in the 3 days before the illness began, the probability of observing this difference in proportions by chance alone is 0.000045, and the ratio of the odds of exposure is 15, a measure of the strong association between disease and consuming sliced mangos. When disease is statistically associated with more than one exposure, multivariate analysis can identify truly independent risk factors. In the above example, "eating food from a street vendor" would not be independent from "eating sliced mango" if one usually got sliced mango from a street vendor. This

Table 1. Cholera cases by country, as reported to the Pan American Health Organization, 1991 through 1994

| | | Number of reported cases | | | |
|---|---|---|---|---|---|
| Country | Date of first report | 1991 | 1992 | 1993 | 1994 |
| Argentina | Feb. 5, 1992 | 0 | 553 | 2,080 | 889 |
| Belize | Jan. 9, 1992 | 0 | 159 | 135 | 6 |
| Bolivia | Aug. 26 1991 | 206 | 22,260 | 10,134 | 2,710 |
| Brazil | Apr. 8, 1991 | 2,101 | 30,054 | 56,286 | 49,455 |
| Chile | Apr. 12, 1991 | 41 | 73 | 32 | 1 |
| Colombia | Mar. 10, 1991 | 11,979 | 15,129 | 230 | 996 |
| Costa Rica | Jan. 3, 1992 | 0 | 12 | 14 | 38 |
| Ecuador | Mar. 1, 1991 | 46,320 | 31,870 | 6,833 | 1,785 |
| El Salvador | Aug. 19, 1992 | 947 | 8,106 | 6,573 | 11,739 |
| French Guiana | Dec. 14, 1992 | 1 | 16 | 2 | NR[a] |
| Guatemala | July 24, 1991 | 3,652 | 15,686 | 30,605 | 4,227 |
| Guyana | Nov. 5, 1992 | 0 | 5 | 66 | 0 |
| Honduras | Oct. 13, 1991 | 17 | 388 | 2,290 | 4,965 |
| Mexico | June 13,1991 | 2,690 | 8,162 | 10,712 | 4,059 |
| Nicaragua | Nov. 12, 1991 | 1 | 3,067 | 6,631 | 7,821 |
| Panama | Sept.10, 1991 | 1,178 | 2,416 | 42 | 0 |
| Paraguay | Jan. 25, 1993 | 0 | 0 | 3 | 0 |
| Peru | Jan. 23, 1991 | 322,562 | 210,836 | 71,448 | 23,887 |
| Suriname | Mar. 6, 1992 | 0 | 2 | 0 | 0 |
| United States | Apr. 9, 1991 | 26 | 103 | 22 | 34 |
| Venezuela | Nov. 29, 1991 | 13 | 2,842 | 409 | 0 |
| Total | | 391,734 | 352,300 | 204,547 | 112,612 |

[a] NR = no reports received.
Source: ref. 2.

case-control method can be rapidly carried out in the field at low cost.

Investigations showed that cholera was being transmitted by several distinct mechanisms. The predominant route of transmission in a given setting depends largely on the degree of sanitation already achieved. Therefore, a multifaceted approach to prevention is needed. Emergency measures, such as advice to boil drinking water or to heat all foods from street vendors, are difficult to sustain because of their inconvenience and high cost. Moreover, the cost of building large-scale water treatment and sanitation systems is extraordinary, estimated at $200 billion for all of Latin America (22). The challenge of cholera prevention lies in devising low-cost alternatives that are both effective and sustainable.

Waterborne transmission was identified in seven of the eight investigations. In three of these, the implicated water came from municipal systems or from tanker trucks that reportedly obtained water from municipal systems. Water delivered through poorly maintained municipal water systems can be contaminated by sewage because of leaky pipes, frequent pressure drops, and the lack of residual chlorine disinfectant in the water. In developing countries, water is rarely available 24 hours a day so it is usually stored in the home, where further contamination can easily occur. For example, when we measured the increase in contamination of the water as it was distributed and stored in Trujillo, Peru, fecal coliform counts, an index of sewage contamination, were 1/100 ml in water collected at the source well, 2/100 ml at public taps, and 20/100 ml in water stored in the home (13) In four investigations, the implicated water was collected from rivers or ponds, where direct sewage contamination was likely. Specific protective practices were also noted in these investigations, including treating water in the home by boiling it or by adding chlorine bleach, using a small-mouthed vessel to store water, pouring water out of the storage vessel rather than scooping it out with a cup, and having hand soap in the home. In one investigation on the Amazon, we found that the local common practice of adding citrus juice to water to improve its taste was protective because the acid in the fruit killed *Vibrio* bacteria (14). This observation gave local authorities a new, inexpensive, and immediately available emergency control measure.

The first stage of prevention, then, is providing safe drinking water. As a result of the above findings, we have developed and are testing simple and inexpensive methods of domestic water disinfection and storage that would also prevent other diseases transmitted by the same route. A pilot trial in a periurban area of Bolivia showed that disinfecting household water with a calcium hypochlorite solution and storing it safely in a special narrow-mouthed container was acceptable to a community of Aymara Indians (20). Compliance,

Table 2. Mechanisms of transmission of epidemic cholera in Latin America, as determined in eight epidemiologic investigations, 1991-1993[a]

| Transmission mechanism | Peru | | | Ecuador | El | Bolivia | Brazil | Guatemala |
|---|---|---|---|---|---|---|---|---|
| | Trujillo Urban 3/91 (15) | Piura Urban 3/91 (16) | Iquitos Urban 7/91 (17) | Guayaquil Urban 7/91 (18) | Salvador Rural 3/91 (19) | Saipina Rural 2/92 (20) | Fortaleza Rural 6/93 +[b] | Guatemala City Urban 7/93 (21) |
| Waterborne | | | | | | | | |
| Municipal water | + | + | | + | | | | |
| Surface water | | | + | | + | + | + | |
| Putting hands in water vessel | + | + | | | | | | |
| Foodborne | | | | | | | | |
| Street vendors' foods | | + | | | | | | + |
| Street vendors' beverages | | + | | + | | | | + |
| Street vendors' ice/ices | | + | | | | | | + |
| Leftover rice | | + | + | | | | | + |
| Fruits/vegetables | | | + | | | | | |
| Seafood | | | | | | | | |
| Uncooked seafood | | | | + | | | | |
| Cooked seafood | | | | + | + | | | |

[a] Reference numbers are in parentheses.
[b] CDC Unpublished data.

as measured by chlorine residuals in stored water, was high among families using the intervention. The concentration of *Escherichia coli* bacteria in the stored water, a measure of fecal contamination, was significantly lower in households that used the intervention than in neighboring households that used traditional water handling methods (23). A field trial in rural Bolivia showed that villagers could generate their own disinfectant solution by using a simple electrolytic apparatus (24); with the disinfectant and the special water container the villagers provided clean water in their homes. Households using this intervention had 40% fewer diarrheal episodes than randomly selected neighboring families who used traditional water-handling methods. The combination of point-of-use disinfection and safer water storage containers could have broad effects, including local empowerment for production of potable water, safer infant foods, and new microindustries for the production of disinfectant solutions and water vessels (25). Cost-benefit analysis indicates that this strategy is cost saving if it prevents more than 20% of diarrheal illnesses (26). Inexpensive disinfectant generators are now being manufactured for this purpose in Ecuador. In Colombia, the number of cholera cases has dropped dramatically since late 1992 when chlorine disinfectant tablets were distributed for treating household water.

The second major route of cholera transmission is food contaminated in the market or home. This includes food and beverages sold by street vendors, leftover rice, and unwashed fruits and vegetables. This was an important route in four of the eight investigated areas, including Guatemala City, where there was no evidence of waterborne transmission (21). Foods and beverages sold by street vendors are a fixture of urban life throughout the developing world; they are often prepared in unhygienic ways and then held at ambient temperatures for hours, which permits rapid bacterial multiplication. Other problems associated with food and beverages from street vendors included using unsafe ice to chill beverages and selling homemade frozen drinks. Leftover rice is an excellent growth medium for *V. cholerae* O1 and eating leftover rice without reheating it was associated with illness in three investigations. In one investigation, illness was associated with eating unwashed produce that was probably splashed with river water while being transported to market in small boats.

Thus, the second stage of cholera prevention is to improve food handling, particularly for foods and beverages sold by street vendors. Many countries in Latin America have begun educating street vendors in fundamental food safety and linking this education to licensing (27). By itself, however, education may not improve food safety if clean water to prepare foods and beverages is not available and handwashing and dishwashing with soap and water are not routine. We are field testing a combined strategy of point-of-use disinfection, handwashing with soap, and use of a special water/beverage container to improve the microbial quality of beverages sold by street vendors. Because street vendors are responsive to customer demand, teaching consumers to look for street vendors that are visibly practicing better hygiene may reinforce more hygienic conditions. In addition to these efforts to improve food sold on the street, health authorities should advise the public to reheat leftover rice and wash fruits and vegetables before eating. In Santiago, Chile, suspicion that cholera was caused by vegetables irrigated with fresh sewage led to a ban on this practice; the ban not only prevented cholera transmission by this route, but also decreased the incidence of typhoid fever and hepatitis A dramatically (28).

Transmission through seafood (identified in two of the eight investigations) is a third major route of cholera transmission, distinct from other foodborne mechanisms, because it requires different prevention strategies. One investigation implicated both uncooked seafood and cooked crab, and another implicated cooked seafood eaten without reheating. Contaminated seafood also caused three outbreaks of travel-associated cholera in the United States. In the most dramatic, at least 75 persons contracted cholera after taking a flight from Latin America to California (29); illness was associated with eating cold seafood salad that was loaded onto the plane in Lima, Peru. Two other outbreaks followed the informal transport of cooked crabs from Ecuador to the United States in travelers' suitcases (30,31). Marine creatures may harbor *V. cholerae* O1 before they are harvested or may be contaminated by seawater used in seaside processing plants. In areas where raw and undercooked seafood are popular, seafood-associated cholera may occur even if the general level of sanitation and hygiene is high (32). Vibrios survive light cooking and can subsequently grow if the seafood is held for many hours before eating (33).

Preventing seafood-associated cholera in the long term will depend on maintaining sewage-free harvest beds and improving sanitation in processing plants. In coastal areas where the organism persists in the environment, even in the absence of sewage contamination, education to discourage the consumption of raw or undercooked shellfish is also needed. Thorough cooking provides the greatest security, but it is sometimes resisted by local populations for cultural reasons. "Ceviche," a popular Latin American dish prepared from seafood that is marinated in citrus juice for variable lengths of time, is a case in point. Prolonged marination in acidic liquid is likely to inactivate vibrios if the acid can penetrate throughout the flesh and deep organs of the fish or shellfish (34). Further evaluation of this approach is needed, but for the present, encouraging the use of ceviche recipes that provide sufficient marination time may be a practical intervention.

In Latin America, as in other parts of the world, epidemiologic field investigations of cholera have defined the local routes of transmission, identified unsuspected and correctable control points, and quantified the effects of emergency measures. The results of investigations also have generated specific control strategies targeted to blocking the predominant routes. While this multistage portrait of transmission is complex, it is being translated into action and change. The longstanding deficits in basic urban infrastructure and the need for new efforts to correct them have never been more apparent (35,36). Workable prevention strategies include better domestic water storage containers, point-of-use water disinfection, attention to the education and hygiene of street vendors, and simple modifications of traditional recipes. Many other diseases are transmitted by these same waterborne and foodborne routes, so these control measures may prevent other infections in addition to cholera. If it becomes a catalyst for long overdue improvements in the safety of water and food, epidemic cholera can have a far-reaching impact on the public health of Latin America.

**Robert V. Tauxe, M.D., M.P.H., Eric D. Mintz, M.D., M.P.H., Robert E. Quick, M.D., M.P.H.**
National Center for Infectious Diseases, Centers for Disease Control and Prevention, Atlanta, Georgia, USA

### References

1. Centers for Disease Control. Cholera—Peru, 1991. MMWR 1991;40:108-9.
2. Pan-American Health Organization. Cholera in the Americas. Epidemiol Bull 1995;16:11-3.
3. Wachsmuth IK, Evins GM, Fields PI, Olsvik Ø, Popovic T, Bopp CA, et al. The molecular epidemiology of cholera in Latin America. J Infect Dis 1993;167:621-6.
4. Evins GM, Cameron DN, Wells JG, Greene KD, Popovic T, Giono-Cerezo S, et al. The emerging diversity of the electrophoretic types of *Vibrio cholerae* in the Western Hemisphere. J Infect Dis 1995;172:173-9.
5. Ramamurthy T, Garg S, Sharma R, Bhattacharya SK, Nair GB, Shimada T, et al. Emergence of a novel strain of *Vibrio cholerae* with epidemic potential in southern and eastern India. Lancet 1993;341:703-4.
6. Mekalanos JJ, Sadoff JC. Cholera vaccines: fighting an ancient scourge. Science 1994;265:1387-9.
7. Sanchez JL, Vasquez B, Begue RE, Meza R, Castellares G, Cabezas C, et al. Protective efficacy of oral whole-cell/recombinant-B-subunit cholera vaccine in Peruvian military recruits. Lancet 1994;344:1273-6.
8. Levine MM, Tacket CO. Recombinant live oral vaccines. In: Wachsmuth IK, Blake PA, and Olsvik Ø, editors. *Vibrio cholerae* and Cholera. Washington, DC: American Society for Microbiology, 1994:395-413.
9. Rosenberg CE. The cholera years: The United States in 1832, 1849, and 1866. Chicago: University of Chicago Press, 1987.
10. Blake PA. Epidemiology of cholera in the Americas. Gastroenterol Clin North Am 1993;22:639-60.
11. Snow J. On the mode of communication of cholera. The Commonwealth Fund. London: Oxford University Press, 1936.
12. Mintz ED, Popovic T, Blake PA. Transmission of *Vibrio cholerae* O1. In: Wachsmuth IK, Blake PA, and Olsvik O, editors. *Vibrio cholerae* and cholera. Washington, DC: American Society for Microbiology, 1994:345-56.
13. Kolvin JL, Roberts D. Studies on the growth of *Vibrio cholerae* biotype El Tor and biotype classical in foods. J Hyg (Cambridge) 1982;89:243-52.
14. Blake PA, Rosenberg ML, Florencia J, Costa JB, Quintino L do P, Gangarosa EJ. Cholera in Portugal, 1974. II. Modes of transmission. Am J Epidemiol 1977;105:344-8.
15. Swerdlow DL, Mintz ED, Rodriguez M, Tejada E, Ocampo C, Espejo L, et al. Waterborne transmission of epidemic cholera in Trujillo, Peru: lessons for a continent at risk. Lancet 1992;340:28-32.
16. Ries AA, Vugia DJ, Beingolea L, Palacios AM, Vasquez E, Wells JG, et al. Cholera in Piura, Peru: a modern urban epidemic. J Infect Dis 1992;166:1429-33.
17. Mujica OJ, Quick RE, Palacios AM, Beingolea L, Vargas R, Moreno D, et al. Epidemic cholera in the Amazon: The role of produce in disease risk and prevention. J Infect Dis 1994;169;1381-4.
18. Weber JT, Mintz ED, Cañizares R, Semiglia A, Gomez I, Sempértegui R, et al. Epidemic cholera in Ecuador: multidrug resistance and transmission by water and seafood. Epidemiol Infect 1994;112:1-11.
19. Quick RE, Thompson BL, Zuniga A, Dominguez G, de Brizuela EL, de Palma O, et al. Epidemic cholera in rural El Salvador: risk factors in a region covered by a cholera prevention campaign. Epidemiol Infect 1995;114:249-55.

20. Gonzales O, Aguilar A, Antunez D, Levine W. An outbreak of cholera in rural Bolivia: rapid identification of a major vehicle of transmission. 32nd Interscience Conference on Antimicrobial Agents and Chemotherapy, Anaheim, 1992. Washington, DC: American Society for Microbiology 1992; Abstract 937.
21. Koo D, Aragon A, Moscoso V, Gudiel M, Bietti L, Carrillo N, et al. Epidemic cholera in Guatemala, 1993: transmission of a newly introduced epidemic strain by street vendors. Epidemiol Infect 1995; (in press).
22. de Macedo CG. Presentation of the PAHO regional plan. Proceedings of the Conference: Confronting cholera, the development of a hemispheric response to the epidemic; 1991 Jul 8-9; Miami: University of Miami, 1991;39-44.
23. Quick R, Venczel L, Gonzales O, Damiani E, Highsmith A, Espada A, et al. Impact of narrow-necked water vessels and home chlorination on fecal coliform and *E. coli* counts in drinking water. 33rd Interscience Conference on Antimicrobial Agents and Chemotherapy, New Orleans, 1993. Washington, DC: American Society for Microbiology, 1993.
24. Quick R, Venczel L, Mintz E, Bopp C, Soleto L, Bean N, et al. Diarrhea prevention in Bolivia through safe water storage vessels and locally-produced mixed oxidant disinfectant. 35th Interscience Conference on Antimicrobial Agents and Chemotherapy, San Francisco, 1995. Washington, DC: American Society for Microbiology, 1995; Abstract 1347.
25. Mintz ED, Reiff FM, Tauxe RV. Safe water treatment and storage in the home: a practical new strategy to prevent waterborne disease. JAMA 1995;273:948-53.
26. Miller M, Quick R, Mintz E, Tauxe R, Teutsch S. Solid stools and solvent citizens: an effective solution for preventing diarrhea in developing countries. 34th Interscience Conference on Antimicrobial Agents and Chemotherapy, Orlando, 1994. Washington, DC: American Society for Microbiology, 1994. Abstract 1244.
27. Arambulo P, Almeida CR, Cuellar J, Bellotto AJ. Street food vending in Latin America. Bull PAHO 1994;28:244-54.
28. Alcayaga S, Alcagaya J, Gassibe P. Changes in the morbidity profile of certain enteric infections after the cholera epidemic. Rev Chil Infect 1993;1:5-10.
29. Centers for Disease Control and Prevention. Cholera associated with an international airline flight, 1992. MMWR 1992;41:134-5.
30. Finelli L, Swerdlow D, Mertz K, Ragazzoni H, Spitalny K. Outbreak of cholera associated with crab brought from an area with epidemic disease. J Infect Dis 1992;166:1433-5.
31. Centers for Disease Control. Cholera—New York, 1991. MMWR 1991;40:516-8.
32. Lowry PW, Pavia AT, McFarland LM, Peltier BH, Barrett TJ, Bradford HB, et al. Cholera in Louisiana: widening spectrum of seafood vehicles. Arch Intern Med 1989;149:2079-84.
33. Blake PA, Allegra DT, Snyder JD, Barrett TJ, McFarland L, Caraway CT, et al. Cholera—a possible endemic focus in the United States. N Engl J Med 1980;302:305-9.
34. Mata L. Efecto del jugo y de la pulpa de frutas acidas sobre el *Vibrio cholerae.* In: El Cólera: Historia, prevención y control. San José, Editorial Universidad Estatal a Distancia - Editorial de la Universidad de Costa Rica, 1992, 275.
35. Sepulveda J, Gomez-Dantes H, Bronfman M. Cholera in the Americas: an overview. Infection 1992;20:243-8.
36. Witt VM, Reiff FM. Environmental health conditions and cholera vulnerability in Latin America and the Caribbean. J Public Health Policy 1991;12:450-63.
